# Genetic landscape in Russian patients with familial left ventricular noncompaction

**DOI:** 10.3389/fcvm.2023.1205787

**Published:** 2023-05-24

**Authors:** Alexey N. Meshkov, Roman P. Myasnikov, Anna V. Kiseleva, Olga V. Kulikova, Evgeniia A. Sotnikova, Maria M. Kudryavtseva, Anastasia A. Zharikova, Sergey N. Koretskiy, Elena A. Mershina, Vasily E. Ramensky, Marija Zaicenoka, Yuri V. Vyatkin, Maria S. Kharlap, Tatiana G. Nikityuk, Valentin E. Sinitsyn, Mikhail G. Divashuk, Vladimir A. Kutsenko, Elena N. Basargina, Vladimir I. Barskiy, Nataliya A. Sdvigova, Olga P. Skirko, Irina A. Efimova, Maria S. Pokrovskaya, Oxana M. Drapkina

**Affiliations:** ^1^ National Medical Research Center for Therapy and Preventive Medicine of the Ministry of Healthcare of the Russian Federation, Moscow, Russia; ^2^ National Medical Research Center for Cardiology of the Ministry of Healthcare of the Russian Federation, Moscow, Russia; ^3^ Hereditary Metabolic Diseases Laboratory, Research Centre for Medical Genetics, Moscow, Russia; ^4^ Department of General and Medical Genetics, Pirogov Russian National Research Medical University, Moscow, Russia; ^5^ Faculty of Bioengineering and Bioinformatics, Lomonosov Moscow State University, Moscow, Russia; ^6^ Medical Research and Educational Center, Lomonosov Moscow State University, Moscow, Russia; ^7^ Phystech School of Biological and Medical Physics, Moscow Institute of Physics and Technology, Dolgoprudny, Russia; ^8^ Department of Natural Sciences, Novosibirsk State University, Novosibirsk, Russia; ^9^ Laboratory of Applied Genomics and Crop Breeding, All-Russia Research Institute of Agricultural Biotechnology, Moscow, Russia; ^10^ Faculty of Mechanics and Mathematics, Lomonosov Moscow State University, Moscow, Russia; ^11^ National Medical Research Center for Children’s Health, Moscow, Russia

**Keywords:** LVNC, left ventricular noncompaction cardiomyopathy, genetic screening, family form, *MYH7*, *TTN*

## Abstract

**Background:**

Left ventricular noncompaction (LVNC) cardiomyopathy is a disorder that can be complicated by heart failure, arrhythmias, thromboembolism, and sudden cardiac death. The aim of this study is to clarify the genetic landscape of LVNC in a large cohort of well-phenotyped Russian patients with LVNC, including 48 families (n=214).

**Methods:**

All index patients underwent clinical examination and genetic analysis, as well as family members who agreed to participate in the clinical study and/or in the genetic testing. The genetic testing included next generation sequencing and genetic classification according to ACMG guidelines.

**Results:**

A total of 55 alleles of 54 pathogenic and likely pathogenic variants in 24 genes were identified, with the largest number in the MYH7 and TTN genes. A significant proportion of variants −8 of 54 (14.8%) −have not been described earlier in other populations and may be specific to LVNC patients in Russia. In LVNC patients, the presence of each subsequent variant is associated with increased odds of having more severe LVNC subtypes than isolated LVNC with preserved ejection fraction. The corresponding odds ratio is 2.77 (1.37 −7.37; p <0.001) per variant after adjustment for sex, age, and family.

**Conclusion:**

Overall, the genetic analysis of LVNC patients, accompanied by cardiomyopathy-related family history analysis, resulted in a high diagnostic yield of 89.6%. These results suggest that genetic screening should be applied to the diagnosis and prognosis of LVNC patients.

## 1. Introduction

Left ventricular noncompaction (LVNC) cardiomyopathy is characterized by the presence of a two-layer structure in the myocardium. Its main layer is represented by a compact myocardium, and the other layer is a spongy structure with multiple trabeculae (1). Patients are diagnosed with LVNC after an echocardiography (ECHO) or cardiac magnetic resonance (CMR) examination (2, 3). LVNC, due to the development of diagnostic methods and increased awareness of the disease, has become more often diagnosed in both children and adults (4). Due to the variety of clinical subtypes of the disease, the tendency to develop life-threatening arrhythmias, sudden cardiac death (SCD), heart failure, and the underestimation of the disease by many clinicians, LVNC is a disease that requires better clinical identification, as well as an understanding of pathology for the timely initiation of therapy (1). Initially, for the diagnosis of LVNC, the identification and description of trabeculations were most crucial, but later other features became important in defining specific LVNC subtypes (5). There are several subtypes of LVNC: isolated, dilated, hypertrophic, and restrictive. The genetic nature of the disease is detected in only half of the cases; however, as sequencing data accumulates, their number will grow (6). About 180 genes associated with LVNC, with varying levels of evidence, were reported (7). For heterogeneous disorders such as LVNC, a better understanding of its genetic background could improve outcome prediction and patient management. The aim of this study was to clarify the genetic landscape of LVNC in a large cohort of well-phenotyped Russian patients listed in the multicenter LVNC register (8).

## 2. Materials and methods

### 2.1. Selection of participants and clinical data

Index patients with LVNC and burdened family history of LVNC and their relatives were included in this study. The inclusion criteria were: the presence of LVNC in an index patient and at least one relative of 1st, 2nd, or 3rd degree with LVNC or other cardiomyopathies. All index patients underwent clinical examination and genetic analysis, as well as family members who agreed to participate in the clinical study and/or in the genetic testing. Clinical examination included general examination, electrocardiography using 24-h Holter monitoring electrocardiogram, CMR, ECHO, and blood sample collection for biochemical and genetic analyses. For ECHO and CMR imaging, the criteria of LVNC suggested by Jenni et al. (2) and Petersen et al. (3) were used. The study was conducted according to the guidelines of the Declaration of Helsinki and approved by the Institutional Review Boards of the National Medical Research Center for Therapy and Preventive Medicine (Moscow, Russia). Every participant and/or their legal representative gave their written informed consent to be involved in this study.

The patients were classified into the 7 subtypes of LVNC: (1) isolated LVNC with preserved ejection fraction (EF) of the left ventricular (LV), if they had normal LV dimensions without LV hypertrophy; (2) isolated LVNC with reduced EF of the LV, if the patient had normal LV dimensions with EF < 50%; (3) dilated LVNC if the patient had LV dilatation; (4) hypertrophic LVNC, if the patient also had LV wall hypertrophy of ≥13 mm; (5) hypertrophic dilated LVNC, if the patient had LV dilation and LV wall hypertrophy; (6) LVNC with congenital heart disease (CHD); (7) restrictive LVNC is characterized by left atrial or biatrial dilation and diastolic dysfunction.

For pedigrees creation, the CeGaT Pedigree Chart Designer v.3.0 (CeGaT GmbH, Tübingen, Germany) was used (9).

### 2.2. Echocardiography and cardiac magnetic resonance

ECHO was performed using the ultrasound system Philips IE33 (Philips Medical Systems, Eindhoven, Netherlands). In the parasternal position on the short axis at the end of the systole were evaluated the presence or absence of a two-layer structure of the myocardium, the presence of trabeculae and blood flow between them, and the ratio of the thickness of the noncompact layer to the thickness of the compact layer were evaluated more than twice (2).

A CMR was performed using a 1.5 T scanner (Avanto, Siemens Medical Solutions, Erlangen, Germany) with retrospective ECG-gating. Standard protocols consisted of breath-hold cine-imaging (SSFP) and late gadolinium enhancement (LGE) were implemented. We analyzed the results using CVI 42 software. LV end-diastolic volume (EDV), LV end-systolic volume (ESV), myocardial mass, and non-compacted myocardial mass were calculated as well as indexed values. Patterns of contrast enhancement were analyzed.

### 2.3. Genetic analysis

#### 2.3.1. DNA extraction

The blood samples and buccal swabs were stored at −30°C and +4°C, respectively, at the Biobank of the National Medical Research Center for Therapy and Preventive Medicine (Moscow, Russia) (10). DNA was extracted from whole blood and, in one case, buccal swab samples using the QIAamp DNA Blood Mini Kit (Qiagen, Hilden, Germany). The DNA concentration was measured with a Qubit 4 fluorometer (Thermo Fisher Scientific, Waltham, MA, USA).

#### 2.3.2. NGS

Next generation sequencing (NGS) was performed with three platforms (HiSeq 1500, NextSeq 550, and Ion S5) for 141 patients, including 48 index patients (see Supplementary Table S1). Sanger sequencing was performed for other relatives. The list of studied genes is presented in Supplementary Table S2. All sequencing stages were carried out in accordance with the manufacturers' protocols.

##### 2.3.2.1. Exome sequencing

Exome sequencing was done on two platforms, HiSeq 1500 and NextSeq 550 (Illumina, San Diego, CA, USA).

For the NextSeq 550 platform, exome libraries were prepared with the TruSeq DNA Library Preparation Kit (Illumina, San Diego, CA, USA) and the xGen Exome Research Panel (IDT, Integrated DNA Technologies, Coralville, IA, USA) according to the IDT-Illumina TruSeq DNA Exome protocol (Illumina, San Diego, CA, USA). Sequencing was done using NextSeq 550 (Illumina, San Diego, CA, USA) with paired-end sequencing (150 bp) (11, 12).

For the HiSeq 1500 platform, exome libraries were prepared using the Kapa Library Amplification Kit (Roche, Basel, Switzerland) and NimbleGen SeqCap EZ Exome v3.0 (Roche, Basel, Switzerland). Sequencing was performed on HiSeq 1500 (Illumina, San Diego, CA, USA) with paired-end sequencing (250 bp).

##### 2.3.2.2. Custom panel sequencing

We developed a list of genes for analysis on the basis of information about association with cardiomyopathies published in the HPO, ClinGen, OMIM, and ClinVar databases (13–16) and literature (17–19). The intersection of this list with the lists of genes included in the panels used in this study is presented in Supplementary Table S2. In the course of exome sequencing, all 297 genes were analyzed. For the two custom panels, 137 and 200 genes, respectively, were analyzed.

For the Ion S5 platform, a custom panel was used. It included 137 genes (CDS + 10 bp padding) associated with LVNC or other cardiomyopathies (Supplementary Table S2) and was designed in the Ion AmpliSeq Designer software (Thermo Fisher Scientific, Waltham, MA, USA). The preparation of AmpliSeq libraries was done on the Ion Chef System (Thermo Fisher Scientific, Waltham, MA, USA), the 200 bp sequencing was performed on the Ion S5 (Thermo Fisher Scientific, Waltham, MA, USA) (20).

For the NextSeq 550 platform, a custom panel was used that included exon sequences of 200 (CDS + 25 bp padding) genes, associated with LVNC or other cardiomyopathies (Supplementary Table S2). The libraries were prepared with the SeqCap EZ Prime Choice Library Kit (Roche, Basel, Switzerland). Sequencing was performed on NextSeq 550 (Illumina, San Diego, CA, USA) with paired-end sequencing (150 or 300 bp) (21).

##### 2.3.2.3. Bioinformatic analysis

For the NextSeq 550 and HiSeq 1500 platforms, the first step of sequencing analysis generated fastq files. The GRCh37 reference genome was chosen for the alignment of paired-end reads. We used the custom-designed pipeline based on GATK 3.8 (22) for data processing and quality control evaluation. ENSEMBL Variant Effect Predictor (23), ClinVar (2021/01/10) (16), gnomAD (v2.1.1) (24), and dbSNP (25) databases were applied for the annotation of single-nucleotide variants and short indels. PLINK v1.90 (26) was used to get identity by state (IBS) values and identity by descent (IBD) proportion to estimate relatedness for all pairs of individuals.

For the Ion S5 platform, sequencing and bioinformatic analysis resulted in bam files. We used the Torrent Server (Thermo Fisher Scientific, Waltham, MA, USA) with default parameters to obtain the vcf files. For the annotation of the vcf files Ion Reporter (Thermo Fisher Scientific, Waltham, MA, USA) with the Annotate Variants analysis tool was applied.

#### 2.3.3. Clinical interpretation

Variants in cardiomyopathy-associated genes (Supplementary Table S2) with allele frequencies <0.005 or missing in the gnomAD database were analyzed (24). The pathogenicity evaluation of the variants was done according to the recommendations of the American College of Medical Genetics and Genomics (27). In this study, the following types of variants are included: pathogenic (P), likely pathogenic (LP), and variant of unknown significance (VUS). For all variants, their presence in the ClinVar database (16) was studied.

#### 2.3.4. Sanger sequencing

Sanger sequencing was performed according to the manufacturer's protocol for the purpose of verifying NGS results. PCR-amplified fragments were purified with ExoSAP-IT (Affymetrix, Santa Clara, CA, USA) and then sequenced on the Applied Biosystem 3500 Genetic Analyzer (Thermo Fisher Scientific, Waltham, MA, USA) using the ABI PRISM BigDye Terminator reagent kit v. 3.1 (Thermo Fisher Scientific, Waltham, MA, USA).

### 2.4. Statistical analysis

Statistical analysis was performed using R software (version 3.5.1). For the representation of continuous variables, median (Me) and interquartile range (Q1; Q3) were used; for categorical variables, absolute numbers and percentages were used. For comparison of continuous variables, the Mann–Whitney *U* test was performed, and the two-sided Fisher's exact test was used for categorical variables. Numbers of pathogenic variants in the groups of patients are presented as mean ± standard deviation. A comparison of the number of variants between groups of patients with different LVNC subtypes was carried out using linear and logistic regressions with mixed effects implemented in the lme4 package (28). We used sex and age as fixed effects and the family variable as a random intercept effect. We considered the differences statistically significant if the *p*-value was <0.05. The diagram was created using the ggplot2 package (29) and the Viridis palette (30).

### 2.5. Limitations of the study

Not all study participants underwent NGS genetic testing. NGS genetic testing was carried out using three panels, each including a different number of genes (from 137 to 297). Moreover, it is possible that age-dependent penetrance may play a role in the clinical picture of LVNC, and it can occur that more relatives will be affected in the future. Functional studies to confirm the causal relationship of variants in the *VCL*, *SLC22A5*, and *FHOD3* genes with LVNC development have not been performed.

## 3. Results

### 3.1. Clinical features of LVNC index patients and their relatives

**Figure 1:**
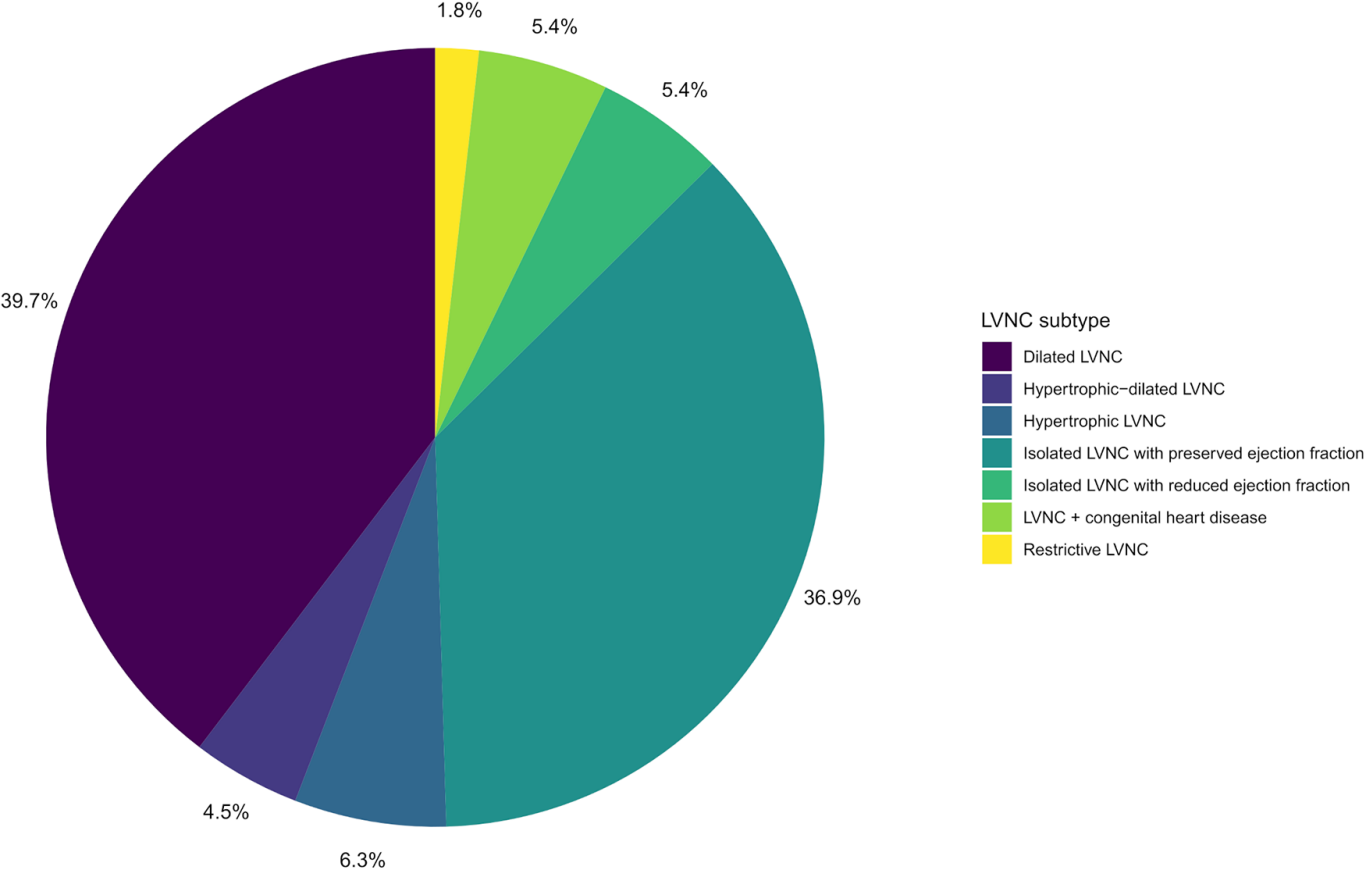
LVNC subtypes diagram of the studied patients.

A total of 48 families with LVNC (48 index patients and 166 relatives) were included in the study. Clinical characteristics of the participants are presented in Table 1. Overall, a total of 121 people had manifestations of LVNC (*n* = 111) or other cardiomyopathies (*n* = 10). Children (persons under 18 years old) and male participants accounted for 29.9% and 48.6% of all participants, respectively. The proportion of men and children did not differ significantly between the groups of patients and healthy people. The group of patients was significantly younger than the group of healthy participants, 32 years vs. 38 years. The LVNC subtypes of the studied patients are shown in the diagram (Figure 1). In individuals with LVNC (*n* = 111), the most common subtypes were dilated and isolated LVNC with preserved EF (76.6% in total). DCM or HCM were diagnosed in 6 and 4 relatives without LVNC, respectively.

**Table 1:** Clinical characteristics of the study participants.

Parameter	All participants (*n* = 214)	LVNC and cardiomyopathy patients (*n* = 121)	Healthy participants (*n* = 93)	*p*-value
Index patients, *n*	48	48	0	—
Relatives, *n*	166	73	93	—
Patients with LVNC subtypes, *n* (%)	111 (51.9)	111 (91.7)	0	—
Patients with other cardiomyopathies	10 (4.7)	10 (8.3)	0	—
Children, *n* (%)	64 (29.9)	38 (29.8)	26 (28)	0.65
Men, *n* (%)	104 (48.6)	64 (52,9)	40 (43)	0.17
Age, years, Me (25; 75)	35.5 (14; 47.3)	32 (13; 42)	38 (17; 56.5)	0.01
BMI, kg/m^2^, >18 y.o., Me (25; 75)	25.8 (22,2; 29.2)	24.9 (21.6; 28.8)	27.3 (23; 31.4)	0.01
EF LV, %, Me (25; 75)	57 (45; 65)	51 (38; 58.5)	63 (60; 68.5)	<0.001
EDV, ml, >18 y.o., Me (25;75)	119.5 (99; 153.3)	135.5 (112; 172.8)	100.5 (87; 125.8)	<0.001
EDD, ml, >18y.o., Me (25;75)	51 (47; 55)	53 (50; 58.8)	48 (45; 51)	<0.001
Nonsustained VT, *n* (%)	30 (16)	30 (26.1)	0	<0.001
Sustained VT *n* (%)	4 (2.3)	4 (3.6)	0	0.07
Thromboembolic event, *n* (%)	4 (2.1)	4 (3.5)	0	0.3
Heart failure, *n* (%)	80 (42.6)	79 (68.7)	1 (1.4)	<0.001
Heart transplantation, *n* (%)	6 (3.1)	6 (5.2)	0	0.08
Neuromuscular diseases, *n* (%)	9 (4.9)	8 (7)	1 (1.4)	0.16
Primary end point, *n* (%)	14 (6.6)	14 (11.7)	0	<0.001
Death, *n* (%)	8 (3.8)	8 (6.7)	0	<0.001
CVD death, *n* (%)	6 (2.8)	6 (5)	0	<0.001
SCD, *n* (%)	2 (0.9)	2 (1.7)	0	<0.001

BMI, body mass index; CVD, cardiovascular disease; EDD, end-diastolic diameter; EDV, end-diastolic volume; EF, ejection fraction; Me, median; SCD, sudden cardiac death; VT, ventricular tachycardia.

### 3.2. Genetic findings in LVNC index patients and their relatives

In 48 index patients, a total of 55 alleles of 54 P/LP variants have been identified in the genes associated with hereditary cardiomyopathies. Results are presented in Table 2, where they are grouped according to the classification given in a recent review (7). Corresponding separately published clinical cases are listed in the reference column. Only one variant was found in two unrelated families (*TPM1*, p.Ala242Val); each of the other variants was found in one family only. The largest number of variants was found in the *MYH7* (27.3%, 15 variants, one of them *de novo* rs730880156) and *TTN* (14.5%, eight variants) genes. We have also identified pathogenic variants in the *VCL* and *SLC22A5* genes that have a limited number of publications (31–33) confirming their association with LVNC but were not listed in the last review of the LVNC genes (6) (see Table 2, Supplementary Table S3). Moreover, we identified one pathogenic variant in the *FHOD3* gene that was previously associated only with hypertrophic cardiomyopathy (HCM) (34–36). No P or LP variants were identified in the five families (10.4%). In 13 index patients, more than one variant was identified (27.1%). Pedigrees of index patients with more than one variant, clinical and genetic information are presented in Supplementary Table S3. In five index patients (10.4%), we additionally identified six rare variants classified as VUS. We suppose that they can modify the course of the disease (see Supplementary Table S4). A total of 14.8% (8 of 54) variants have not been described earlier in other populations and may be specific to LVNC patients in Russia: *DSP*:p.Gln948LysfsTer29, *MYH7*:p.Glu632Lys, *MYH7*:p.Glu497Lys, *TTN*:p.Gln23676HisfsTer16, *ACTN2*:p.Ile190Ser, *ACTN2*: p.Leu70del, *ACTN2*:p.Leu184Pro, *SLC22A5*:p.Asp388lfs*11.

**Table 2:** List of the variants associated with LVNC found in the studied families.

Index patient	Gene	Pathogenicity	Variant (GRCh37)	dbSNP^1^	Consequence^1^	HGVSc^1^	HGVSp^1^	gnomADe AF (NFE) (24)	References^2^
Definitive^3^									
Fam017	*ACTC1*	LP	chr15:35084293A > G	rs397517071	missense_variant, splice_region_variant	ENST00000290378.4:c.806T > C	ENSP00000290378.4:p.Ile269Thr	—	
Fam010	*DES*	LP	chr2:220283520_220283528del	rs1553603239	inframe_deletion	ENST00000373960.3:c.336_344del	ENSP00000363071.3:p.Gln113_Leu115del	—	(37, 38)
Fam023	*DES*	LP	chr2:220285661G > C	rs59962885	missense_variant	ENST00000373960.3:c.1009G > C	ENSP00000363071.3:p.Ala337Pro	—	(20)
Fam023	*DSP*	LP	chr6:7580465del	—	frameshift_variant	ENST00000379802.3:c.4042del	ENSP00000369129.3:p.Leu1348Ter	—	(20)
Fam042	*DSP*	LP	chr6:7580777A > T	—	stop_gained	ENST00000379802.3:c.4354A > T	ENSP00000369129.3:p.Arg1452Ter	—	(39)
Fam042	*DSP*	LP	chr6:7581427dup	—	frameshift_variant	ENST00000379802.3:c.5004dup	ENSP00000369129.3:p.Leu1669ThrfsTer15	—	(39)
Fam306	*DSP*	P	chr6:7577240del*	—	frameshift_variant	ENST00000379802.3:c.2842del	ENSP00000369129.3:p.Gln948LysfsTer29	—	
Fam031	*MIB1*	P	chr18:19378128C > A	rs748226232	stop_gained	ENST00000261537.6:c.1176C > A	ENSP00000261537.6:p.Tyr392Ter	0.000008803	
Fam005	*MYBPC3*	P	chr11:47353740G > A	rs397516037	stop_gained	ENST00000545968.1:c.3697C > T	ENSP00000442795.1:p.Gln1233Ter	0.00001770	(40)
Fam045	*MYBPC3*	P	chr11:47356592C > T	rs397515991	splice_donor_variant	ENST00000545968.1:c.2905 + 1G > A	—	—	
Fam103	*MYBPC3*	LP	chr11:47363542C > T	rs727503195	missense_variant, splice_region_variant	ENST00000545968.1:c.1790G > A	ENSP00000442795.1:p.Arg597Gln	0	
Fam001	*MYH7*	LP	chr14:23887575T > G	—	missense_variant	ENST00000355349.3:c.4013A > C	ENSP00000347507.3:p.His1338Pro	—	(41)
Fam002	*MYH7*	LP	chr14:23894202G > A	rs1064793206	missense_variant	ENST00000355349.3:c.2455C > T	ENSP00000347507.3:p.Arg819Trp	—	
Fam003	*MYH7*	LP	chr14:23894584C > T	—	missense_variant	ENST00000355349.3:c.2330G > A	ENSP00000347507.3:p.Arg777Lys	—	(39)
Fam007	*MYH7*	LP	chr14:23899016C > T	rs397516089	missense_variant	ENST00000355349.3:c.1106G > A	ENSP00000347507.3:p.Arg369Gln	—	
Fam018	*MYH7*	LP	chr14:23897054G > A	—	missense_variant	ENST00000355349.3:c.1628C > T	ENSP00000347507.3:p.Ala543Val	—	(39)
Fam022	*MYH7*	LP	chr14:23887513G > A	rs45451303	missense_variant	ENST00000355349.3:c.4075C > T	ENSP00000347507.3:p.Arg1359Cys	0.000008793	
Fam025	*MYH7*	LP	chr14:23901007A > G	rs397516258	missense_variant	ENST00000355349.3:c.602T > C	ENSP00000347507.3:p.Ile201Thr	—	
Fam027	*MYH7*	LP	chr14:23896511C > T*	—	missense_variant	ENST00000355349.3:c.1894G > A	ENSP00000347507.3:p.Glu632Lys	—	
Fam031^4^	*MYH7*	LP	chr14:23901077C > T	rs730880156	missense_variant, splice_region_variant	ENST00000355349.3:c.532G > A	ENSP00000347507.3:p.Gly178Arg	0	
Fam034	*MYH7*	LP	chr14:23897798C > T*	—	missense_variant	ENST00000355349.3:c.1489G > A	ENSP00000347507.3:p.Glu497Lys	—	
Fam049	*MYH7*	LP	chr14:23888432G > A	—	missense_variant	ENST00000355349.3:c.3926C > T	ENSP00000347507.3:p.Thr1309Ile	—	(39)
Fam056	*MYH7*	LP	chr14:23900824C > G	—	missense_variant	ENST00000355349.3:c.702G > C	ENSP00000347507.3:p.Lys234Asn	—	
Fam062	*MYH7*	LP	chr14:23885272C > T	rs565663412	missense_variant	ENST00000355349.3:c.4894G > A	ENSP00000347507.3:p.Ala1632Thr	0.00002638	(39)
Fam122	*MYH7*	LP	chr14:23900109C > T	rs111547156	splice donor	NM_000257.4:c.895 + 1G > A	—	—	(42)
Fam135	*MYH7*	LP	chr14:23893175C > T	rs886039204	missense_variant	ENST00000355349.3:c.2863G > A	ENSP00000347507.3:p.Asp955Asn	0.000008790	
Fam013, Fam242	*TPM1*	LP	chr15:63354797C > T	rs397516387	missense_variant	ENST00000358278.3:c.725C > T	ENSP00000351022.3:p.Ala242Val	0.000008792	(43)
Fam009	*TTN*	P	chr2:179406990C > G	rs727505319	splice_donor_variant	ENST00000589042.1:c.97492 + 1G > C	—	—	
Fam017	*TTN*	P	chr2:179463948G > A	rs745376275	stop_gained	ENST00000589042.1:c.56572C > T	ENSP00000467141.1:p.Arg18858Ter	0.00001110	(39)
Fam019	*TTN*	P	chr2:179477578G > A	rs1471414348	stop_gained	ENST00000589042.1:c.49870C > T	ENSP00000467141.1:p.Arg16624Ter	—	(44)
Fam021	*TTN*	P	chr2:179439830_179439830delinsG*	—	frameshift_variant	ENST00000589042.1:c.71028_71029delinsC	ENSP00000467141.1:p.Gln23676HisfsTer16	—	
Fam022	*TTN*	LP	chr2:179449453G > A	rs1432889079	stop_gained	ENST00000589042.1:c.64915C > T	ENSP00000467141.1:p.Arg21639Ter	—	
Fam062	*TTN*	P	chr2:179478953G > A	rs570046043	stop_gained	ENST00000589042.1:c.49171C > T	ENSP00000467141.1:p.Arg16391Ter	—	
Fam070	*TTN*	P	chr2:179604264G > A	rs775072385	stop_gained	ENST00000589042.1:c.13696C > T	ENSP00000467141.1:p.Gln4566Ter	0.000008909	
Fam089	*TTN*	LP	chr2:179397983del	rs760768093	frameshift_variant	ENST00000589042.1:c.103360del	ENSP00000467141.1:p.Glu34454AsnfsTer3	0.00004446	
Moderate^3^									
Fam029	*ALPK3*	LP	chr15:85406781A > C	rs1963971731	splice_acceptor_variant	ENST00000258888.6:c.4411-2A > C	—	—	
Fam004	*PRDM16*	P	chr1:3329197del	—	frameshift_variant	ENST00000270722.5:c.2436del	ENSP00000270722.5:p.Ala813ProfsTer58	—	(45)
Fam006	*RBM20*	LP	chr10:112572068C > T	rs267607003	missense_variant	ENST00000369519.3:c.1913C > T	ENSP00000358532.3:p.Pro638Leu	—	(46)
Fam033	*RBM20*	LP	chr10:112581114G > A	rs397516607	missense_variant	ENST00000369519.3:c.2737G > A	ENSP00000358532.3:p.Glu913Lys	—	
Fam008	*TBX20*	LP	chr7:35271175_35271176dup	—	frameshift_variant	ENST00000408931.3:c.830_831dup	ENSP00000386170.3:p.Asp278Ter	—	(11, 39, 47)
Fam064	*TNNT2*	LP	chr1:201331109_201331111del	rs45578238	inframe_deletion	ENST00000509001.1:c.629_631del	ENSP00000422031.1:p.Lys210del	—	
Limited^3^									
Fam037	*ACTN2*	LP	chr1:236891010T > G*	—	missense_variant	ENST00000366578.4:c.569T > G	ENSP00000355537.4:p.Ile190Ser	—	
Fam131	*ACTN2*	LP	chr1:236881238_236881240del*	—	inframe_deletion	ENST00000366578.4:c.207_209del	ENSP00000355537.4:p.Leu70del	—	
Fam132	*ACTN2*	LP	chr1:236890992T > C*	—	missense_variant	ENST00000366578.4:c.551T > C	ENSP00000355537.4:p.Leu184Pro	—	
Fam008	*DSG2*	P	chr18:29111023C > A	rs751527714	stop_gained	ENST00000261590.8:c.1088C > A	ENSP00000261590.8:p.Ser363Ter	0	(11, 39)
Fam024	*FBN2*	LP	chr5:127614327C > T	rs759198660	missense_variant, splice_region_variant	ENST00000508053.1:c.7345G > A	ENSP00000424571.1:p.Asp2449Asn	0.00003548	
Fam011	*FLNC*	LP	chr7:128481344A > C	rs1554398369	missense_variant	ENST00000325888.8:c.1934A > C	ENSP00000327145.8:p.Asp645Ala	—	(48)
Fam031	*MTMR14*	LP	chr3:9695454G > A	rs1413765461	splice_donor_variant	ENST00000296003.4:c.308 + 1G > A	—	0	
Fam003	*MYL2*	LP	chr12:111352058A > G	—	missense_variant	ENST00000228841.8:c.206T > C	ENSP00000228841.7:p.Met69Thr	—	(39)
Fam024	*PLEC*	P	chr8:144994943_144994944del	rs782329610	frameshift_variant	ENST00000322810.4:c.9458_9459del	ENSP00000323856.4:p.Val3153AlafsTer77	0	
Fam245	*PRKAG2*	LP	chr7:151273498C > T	rs121908987	missense_variant	ENST00000287878.4:c.905G > A	ENSP00000287878.3:p.Arg302Gln	—	
Genes, that may indicate an association with LVNC									
Fam026	*FHOD3*	P	chr18:34232893G > A	rs2036163874	splice_donor_variant	ENST00000590592.6:c.1646 + 1G > A	—	—	(49)
Fam003	*VCL*	P	chr10:75855578C > T	rs794729191	stop_gained	ENST00000211998.4:c.1708C > T	ENSP00000211998.4:p.Arg570Ter	0.000008796	(39, 50)
Fam062	*SLC22A5*	P	chr5:131726419del*	—	frameshift_variant	ENST00000245407.3:c.1090del	ENSP00000245407.3:p.Asp364IlefsTer11	—	

^1^ dbSNP identifiers (database version 153), HGVS and consequences are assigned by ENSEMBL VEP.

^2^ References to the more detailed description of the clinical case.

^3^ According to the study (7).

^4^ *de novo* variant, the relationship between index patient and parents was confirmed.

* The variants that have not been described earlier; AF, allele frequency; LP, likely pathogenic; NFE, non-finnish European; P, pathogenic.

Patients with isolated LVNC with preserved EF had 0.74 ± 0.59 causal variants vs. 1.16 ± 0.63 causal variants in patients with other LVNC subtypes. The difference in average numbers of causal variants is equal to 0.42 (0.17–0.66; *p* = 0.001) and decreases to 0.38 (0.18–0.57; *p* < 0.001) after adjustment for sex, age, and family. In patients with LVNC, the presence of each subsequent variant is associated with increased odds of having more severe LVNC subtypes than isolated LVNC with preserved EF. The corresponding odds ratio after adjustments is 3.59 (1.64–10.23; *p* = 0.001) per variant.

## 4. Discussion

The presented work is the largest study on LVNC genetics in Russia in terms of the number of included patients and is comparable with other studies in this field worldwide (6, 17, 51).

This study resulted in several remarkable findings. First of all, genetic analysis in 48 index patients with familial form of LVNC resulted in the diagnostic yield of nearly 90%: 10.4% of index patients had no LP/P variants, 62.5% had one LP/P variant, and 27.1% had more than one variant. This can be compared to other studies, where the percentage of index patients with the familial form of LVNC and no detected pathogenic variants ranged from 34% to 52% (6, 17, 52). In contrast, the absence of a family history of LVNC (sporadic cases) is usually associated with a low diagnostic yield – from 9% to 46% (17, 51). In our study, we used large gene panels containing 137, 200, and 297 genes, which was also the reason for the high diagnostic yield, apparently because it increases with the number of genes for which the analysis of variants is carried out (5, 53). Besides, 27.1% of the studied index patients had more than one variant, which is somewhat higher than in the other studies, e.g., 9.5% in Richard et al. (17) and 16.1% in Miszalski-Jamka et al. (31). This can also be explained by the number of genes analyzed.

Moreover, the high genetic heterogeneity of LVNC in Russia was shown: 54 variants were located in 24 genes, 8 (14.8%) of them were new, and only one variant was found in two unrelated families; each of the other variants was found in one family only. The heterogeneity of LVNC can be confirmed by other studies (5, 54). In our study, the most prevalent genes were *MYH7* (27.3% of variants or 31.3% of index patients) and *TTN* (14.5% of variants or 16.7% of index patients), which is quite common for LVNC studies (17, 31, 52, 55). The next most prevalent genes were *DSP*, *MYBPC3*, *ACTN2*. Most of the 15 variants found in *MYH7* were located in the myosin motor domain (53.3%, eight variants) and in the coiled coil region (33.3%, five variants) (56). Among the eight *TTN* variants, six (75%) were found in the A-band region, one (12.5%) in the I-band region, and one (12.5%) in the M-band region (57), which is similar to other studies (17). The fraction of 76.4% of all variants is located in genes from the “definitive” and “moderate” categories according to the Rojanasopondist et al. classification (7), which indicates the need to prioritize these genes for the genetic diagnostics. At the same time, given the great genetic heterogeneity of LVNC, it may be necessary to evaluate not only the genes from the “limited” category but also the genes associated with other monogenic cardiomyopathies (7).

Here we have described three pathogenic variants in the *VCL*, *SLC22A5*, and *FHOD3* genes. These three cases are rather interesting because variants in other genes that have stronger evidence of association with LVNC did not show full family segregation. From our point of view, it is important to report and describe these cases, which can help clarify the association of these particular genes with LVNC in the future (see Table 2, Supplementary Table S3). The variants in *VCL*, *SLC22A5*, and *FHOD3* have been shown earlier to be associated with HCM or dilated cardiomyopathies (DCM) (13–16, 34–36, 58–61). In the case of *VCL* and *SLC22A5*, there exist only three publications confirming their association with LVNC (31–33). These data support the overlap of genetic pathogenesis between the various cardiomyopathies.

The *VCL* gene codes for vinculin, a membrane-cytoskeletal actin-binding protein. In this study, one family with LVNC had a pathogenic *VCL* variant (p.Arg570Ter) in the index patient, her mother, and sister (ACMG criteria: PVS1, PM2, PP1), two LP variants—*MYH7*:p.Arg777Lys (PM1, PM2, PP2, PP3) and *MYL2*:p.Met69Thr (PM1, PM2, PP2, PP3)—were found in the index patient, her sister, and their healthy father (see Supplementary Table S3). We can only suggest that these LP variants can modify the course of the disease, because the mother of the index patient has a less severe LVNC subtype, but the variant that segregates in all family members with LVNC is in the *VCL* gene.

The *SLC22A5* gene (also known as *OCTN2*) codes for the organic cation transporter novel 2 and is associated with the development of primary carnitine deficiency and cardiomyopathy (62, 63). Here we present a family with the index patient diagnosed with LVNC and three family members diagnosed with DCM (see Supplementary Table S3). Unfortunately, we did not have the opportunity to perform genetic testing for the index patient's husband and sister. Genetic analysis showed that the index patient with LVNC had the LP variant *SLC22A5*:p.D388lfs*11 (ACMG criteria: PVS1, PM2), and that the patient's daughter with DCM is homozygous for this variant. The index patient also had one LP variant *MYH7*:p.Ala1632Thr (PM1, PM2, PP2, PP3) and one P variant *TTN*:p.Arg16391Ter (PVS1, PM2, PP5).

The *FHOD3* gene plays a role in the regulation of the actin cytoskeleton. The clinical description of the *FHOD3* variant clinical case was published earlier (49). In this case, the CMR data allowed for an update of the diagnosis of the index patient and his sister from HCM to LVNC (49).

We believe that the expansion of our knowledge about new genes and variants associated with LVNC will help increase the effectiveness of genetic testing for this disease.

Another finding is related to the risk prediction of the LVNC subtype and associated risk of adverse events, which is important for counseling relatives of patients with LVNC. In this work, the most common LVNC subtypes were dilated LVNC (*n* = 44, 39.6%) and isolated LVNC with preserved EF (*n* = 41, 36.9%). These two subtypes seem to reflect the natural course of the disease and are stages in the same pathogenetic process. In the study by van Waning et al. (52), the share of isolated and dilated LVNC was even higher and amounted to 95%. In the work by Hirono et al., the proportion of these two subtypes was 67% (5).

Different subtypes of LVNC may occur in the same family, and the severity of the disease subtypes may be due to the presence of several variants in the patient (64, 65). Here, we have also shown that the presence of multiple pathogenic variants in one patient is accompanied by the presence of a more severe LVNC subtype. The presence of neuromuscular diseases can also influence the severity of LVNC. In this work, we observed several families with neuromuscular diseases (*n* = 6) who had severe LVNC with progressive heart failure, orthotopic heart transplantation, and fatal outcomes (in 2 cases). In the systematic review by Hirono and Ichida, it was noted that neuromuscular disorders were present in an average of 5% of LVNC patients, and apart from lower EF LV, concomitant neuromuscular disease and heart failure with LV dilation also led to poor prognosis and increased mortality (54). It is worth mentioning that in the group of patients with *MYH7* variants, there were no deaths or orthotopic transplants, which is also consistent with the results of van Waning et al. (6). Besides, patients with CHD had LP variants in *MYH7* or *ACTN2*, while in one of the previous studies only *MYH7* variants were found in such patients (52). Another difference with the study by van Waning et al. is the presence of *TTN* variants among pediatric patients (see Table 2: Fam21, Fam70, and Fam89) (52).

In conclusion, we would like to summarize our findings. The genetic analysis in LVNC patients and the family history of LVNC have a high diagnostic yield. A total of 55 P or LP variants were identified in 44 index patients, with the largest number in the *MYH7* and *TTN* genes. A total of 8 (14.8%) variants were new. Three pathogenic variants in the *VCL*, *SLC22A5*, and *FHOD3* genes were identified, which may indicate an association with LVNC. Different LVNC subtypes may occur in the same family, and the severity of the phenotype may be due to the presence of several variants in the patient. These results support the idea that genetic screening should be applied to the diagnosis and prognosis of LVNC patients.

## Data Availability

The data presented in this study are available on request from the corresponding authors. Individual genotype information cannot be made available in order to protect participant privacy.
