# Supplementary material for: Genetic landscape in Russian patients with familial left ventricular noncompaction
Source: Front Cardiovasc Med. 2023 May 24;10:1205787. doi: 10.3389/fcvm.2023.1205787 (PMC10278580; doi:10.3389/fcvm.2023.1205787)
Supplement: Supplementary file 2 [file Table3.pdf]

Supplementary Table S3. Pedigrees of the index patients with more than one pathogenic variant or, in addition to one pathogenic variant, a rare VUS variant that can modify the cause of the disease.

Pedigree of the index patient Fam003

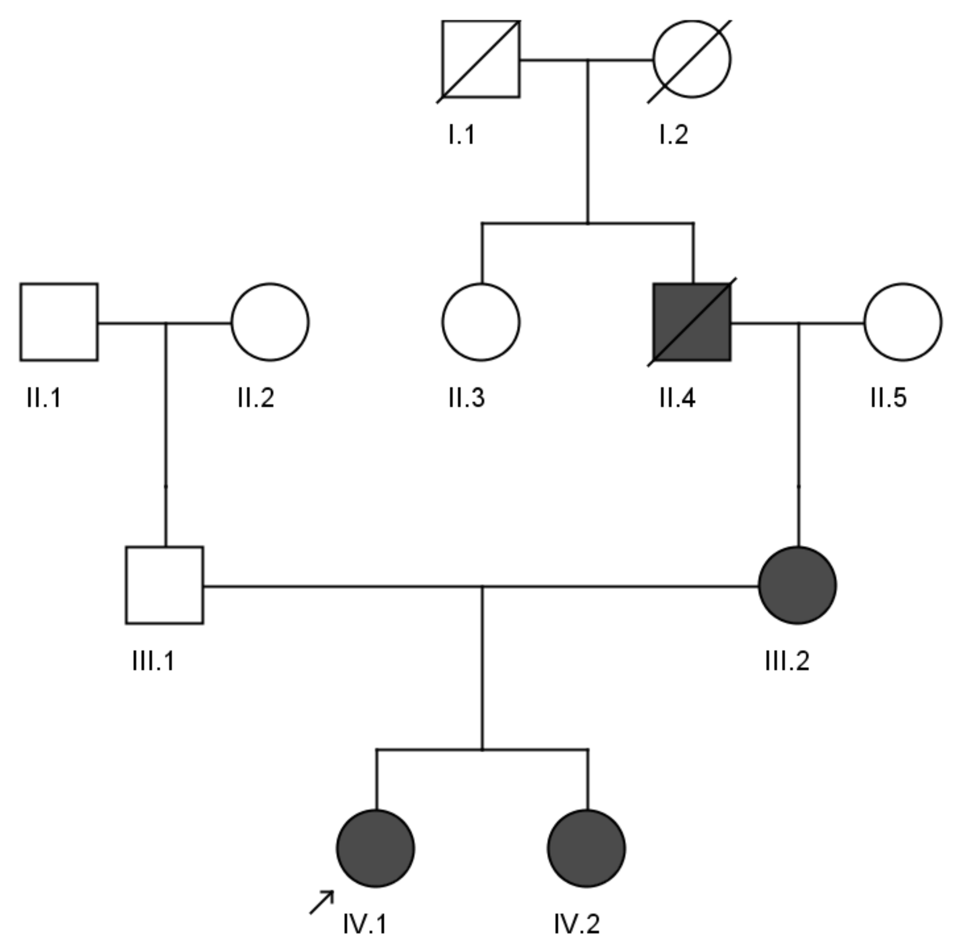

Patient ID	Genotype[1] *VCL*,p.Arg570Ter	Genotype[1] *MYH7*, p.Arg777Lys	Genotype[1] *MYL2*, p.Met69Thr	Genotype[1]	Sex	Age, y.o.	LVNC subtype or other cardiomyopathy	EF, %	Myopathy	NYHA class	Arrhythmia	Conduction disturbance	Outcome
I.1	NA	NA	NA		M	80	NA	NA	NA	NA	NA	NA	died (unknown)
I.2	NA	NA	NA		F	72	NA	NA	NA	NA	NA	NA	died (unknown)
II.1	NA	NA	NA		M	70	NA	NA	NA	NA	NA	NA	alive
II.2	NA	NA	NA		F	69	NA	NA	NA	NA	NA	NA	alive
II.3	NA	NA	NA		F	69	NA	NA	NA	NA	NA	NA	alive
II.4	NA	NA	NA		M	59	NA	NA	no	no	no	no	SCD
II.5	0	0	0		F	66	healthy	62	no	no	no	no	alive
III.1	0	1	1		M	37	healthy	55	no	no	no	no	alive
III.2	1	0	0		F	37	isolated LVNC with preserved EF	57	no	no	VE	no	alive
IV.1	1	1	1		F	9	dilated LVNC	66	no	no	no	no	alive
IV.2	1	1	1		F	5	dilated LVNC	21	no	II	no	no	alive

Pedigree of the index patient Fam008

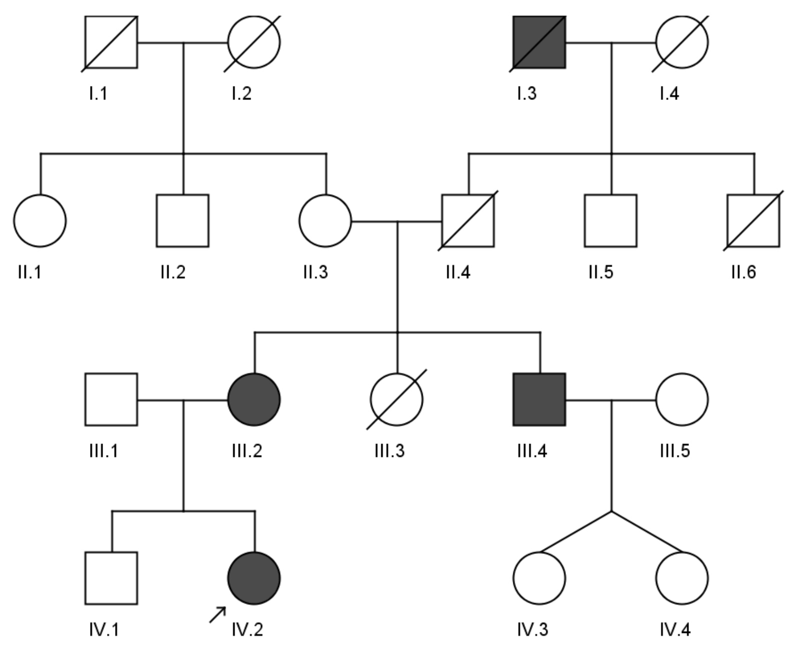

Patient ID	Genotype[1] *DSG2*, p.Ser363Ter	Genotype[1] *TBX20*, p.Asp278Ter	Genotype[1]	Genotype[1]	Sex	Age, y.o.	LVNC subtype or other cardiomyopathy	EF, %	Myopathy	NYHA class	Arrhythmia	Conduction disturbance	Outcome
I.1	NA	NA			M	74	NA	NA	NA	NA	NA	NA	died (unknown)
I.2	NA	NA			F	80	NA	NA	NA	NA	NA	NA	died (unknown)
I.3	NA	NA			M	50	NA	NA	NA	NA	NA	NA	died (cardiomyopathy)
I.4	NA	NA			F	65	NA	NA	NA	NA	NA	NA	died (unknown)
II.1	NA	1			F	72	healthy	62	no	no	no	no	alive
II.2	NA	0			M	68	healthy	58	no	no	no	no	alive
II.3	0	1			F	63	healthy	64	no	no	no	no	alive
II.4	NA	NA			M	57	NA	NA	NA	NA	NA	NA	died (cancer)
II.5	0	0			M	60	healthy	64	no	no	no	no	alive
II.6	NA	NA			M	2	NA	NA	NA	NA	NA	NA	died (unknown)
III.1	0	0			M	42	healthy	61	no	no	no	no	alive
III.2	1	1			F	40	dilated LVNC	42	no	II	no	no	alive
III.3	NA	NA			F	1	NA	NA	NA	NA	NA	NA	died (unknown)
III.4	1	1			M	36	isolated LVNC with preserved EF	56	no	no	no	no	alive
III.5	NA	NA			F	33	healthy	63	no	no	no	no	alive
IV.1	0	0			M	17	healthy	65	no	no	no	no	alive
IV.2	1	1			F	15	dilated LVNC	40	no	II	no	no	alive
IV.3	0	1			F	1	healthy	58	no	no	no	no	alive
IV.4	0	1			F	1	healthy	56	no	no	no	no	alive

Pedigree of the index patient Fam009

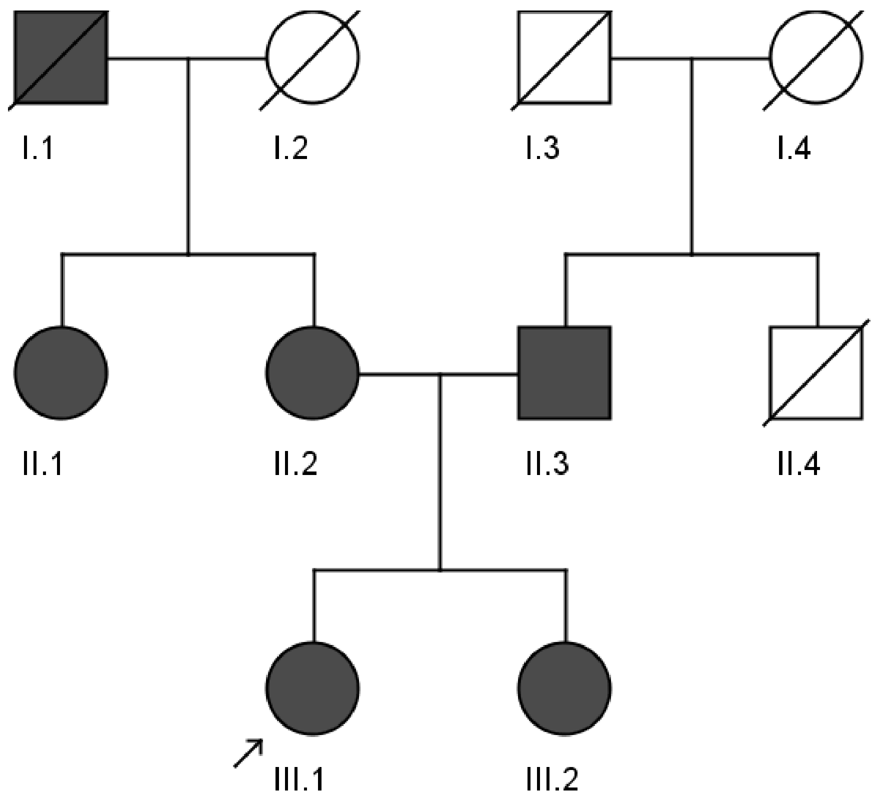

Patient ID	Genotype [1] *TTN*, c.97492+1G>C	Genotype[1] *MYBPC3*, p.Asn782Ser[2]	Genotype[1]	Genotype[1]	Sex	Age, y.o.	LVNC subtype or other cardiomyopathy	EF, %	Myopathy	NYHA class	Arrhythmia	Conduction disturbance	Outcome
I.1	NA	NA			M	51	NA	NA	NA	NA	NA	NA	died (HF)
I.2	NA	NA			F	56	NA	NA	NA	NA	NA	NA	died (cancer)
I.3	NA	NA			M	65	NA	NA	NA	NA	NA	NA	died (unknown)
I.4	NA	NA			F	60	NA	NA	NA	NA	NA	NA	died (cancer)
II.1	1	0			F	61	DCM	45	no	II	AF	no	alive
II.2	1	0			F	53	isolated LVNC with preserved EF	57	no	I	no	no	alive
II.3	0	1			M	60	isolated LVNC with preserved EF	59	no	I	no	no	alive
II.4	NA	NA			M	30	NA	NA	NA	NA	NA	NA	died (accident)
III.1	1	1			F	32	dilated LVNC	18	no	III	VT	no	alive
III.2	0	1			F	31	isolated LVNC with preserved EF	67	no	no	no	no	alive

Pedigree of the index patient Fam013

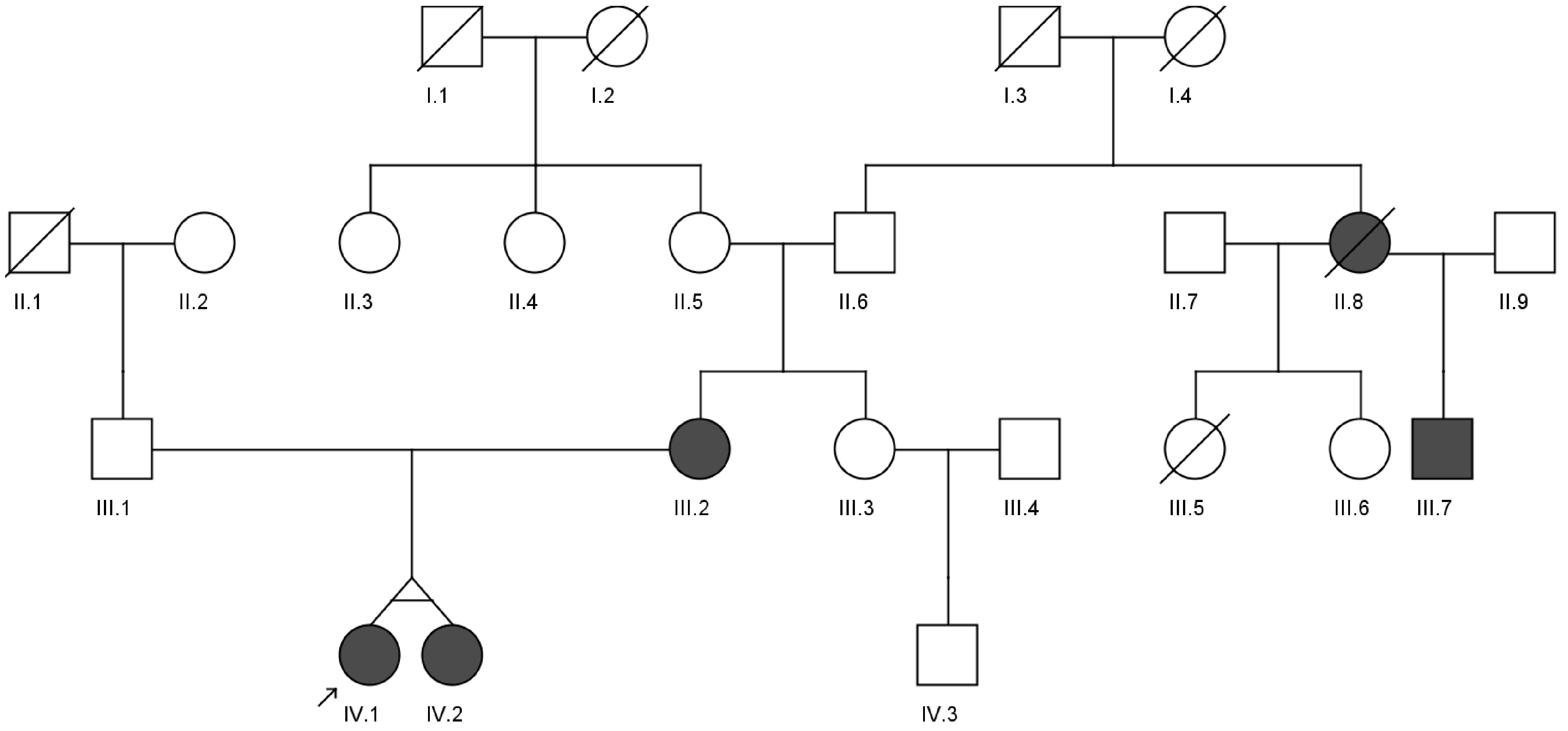

Patient ID	Genotype[1] *TPM1*, p.Ala242Val	Genotype[1] *LDB3*, p.Val307Ile[2]	Genotype[1]	Genotype[1]	Sex	Age, y.o.	LVNC subtype or other cardiomyopathy	EF, %	Myopathy	NYHA class	Arrhythmia	Conduction disturbance	Outcome
I.1	NA	NA			M	62	NA	NA	NA	NA	NA	NA	died (unknown)
I.2	NA	NA			F	72	NA	NA	NA	NA	NA	NA	died (unknown)
I.3	NA	NA			M	63	NA	NA	NA	NA	NA	NA	died (unknown)
I.4	NA	NA			F	87	NA	NA	NA	NA	NA	NA	died (unknown)
II.1	NA	NA			M	67	NA	NA	NA	NA	NA	NA	died (unknown)
II.2	NA	NA			F	70	NA	NA	NA	NA	NA	NA	alive
II.3	NA	NA			F	65	NA	NA	NA	NA	NA	NA	alive
II.4	NA	NA			F	63	NA	NA	NA	NA	NA	NA	alive
II.5	0	NA			F	57	healthy	65	no	no	no	no	alive
II.6	1	NA			M	56	NA	NA	NA	NA	NA	NA	alive
II.7	NA	NA			M	NA	NA	NA	NA	NA	NA	NA	alive
II.8	NA	NA			F	37	DCM	NA	NA	NA	NA	NA	died (DCM, HF)
II.9	NA	NA			M	NA	NA	NA	NA	NA	NA	NA	alive

III.1	0	1			M	48	healthy	63	no	no	no	no	alive
III.2	1	0			F	39	isolated LVNC with preserved EF	64	no	no	no	no	alive
III.3	0	NA			F	32	healthy	56	NA	NA	NA	NA	alive
III.4	NA	NA			M	NA	NA	NA	NA	NA	NA	NA	alive
III.5	NA	NA			F	7	NA	NA	NA	NA	NA	NA	SCD
III.6	NA	NA			F	24	NA	NA	NA	NA	NA	NA	alive
III.7	1	NA			M	19	dilated LVNC	38	no	II	VT	no	alive
IV.1	1	1			F	9	dilated LVNC	43	no	II	no	no	alive
IV.2	1	1			F	9	isolated LVNC with preserved EF	51	no	no	no	no	alive
IV.3	0	NA			M	4	healthy	62	no	no	no	no	alive

Pedigree of the index patient Fam017

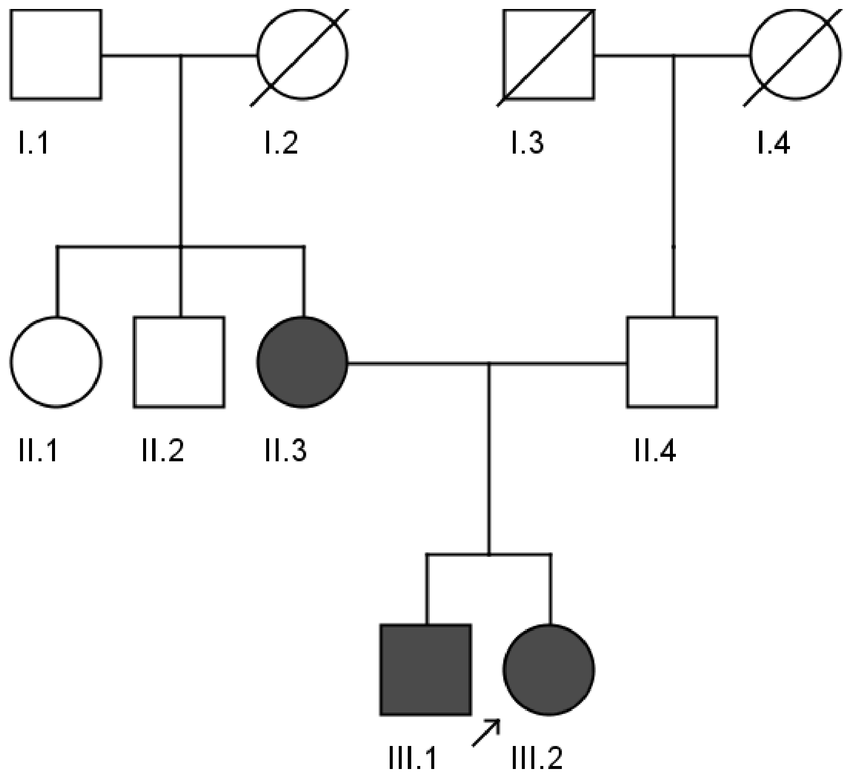

Patient ID	Genotype[1] *TTN*, p.Arg18858Ter	Genotype [1] *ACTC1*, p.Ile269Thr	Genotype [1]	Genotype[1]	Sex	Age, y.o.	LVNC subtype or other cardiomyopathy	EF, %	Myopathy	NYHA class	Arrhythmia	Conduction disturbance	Outcome
I.1	NA	NA			M	67	no	64	no	no	no	no	alive
I.2	NA	NA			F	51	NA	NA	NA	no	AF	no	SCD
I.3	NA	NA			M	65	NA	NA	NA	NA	NA	NA	died (unknown)
I.4	NA	NA			F	64	NA	NA	NA	NA	NA	NA	died (unknown)
II.1	NA	NA			F	51	NA	NA	NA	NA	NA	NA	alive
II.2	NA	NA			M	48	NA	NA	NA	NA	NA	NA	alive
II.3	1	1			F	43	isolated LVNC with preserved EF	42	no	II	VE	no	alive
II.4	NA	NA			M	47	no	65	no	no	no	no	alive
III.1	1	1			M	22	dilated LVNC	38	no	III	VE	no	alive
III.2	1	1			F	11	dilated LVNC	36	no	III	VE	no	alive

Pedigree of the index patient Fam018

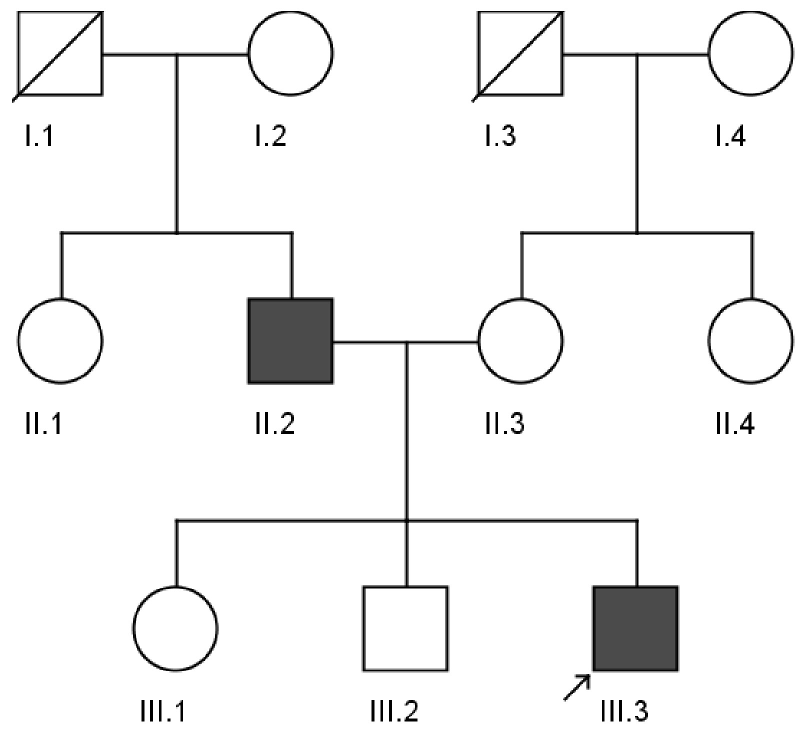

Patient ID	Genotype[1] *MYH7*, p.Ala543Val	Genotype[1] *TBX1*, p.Pro47_Pro48del[2]	Genotype[1]	Genotype[1]	Sex	Age, y.o.	LVNC subtype or other cardiomyopathy	EF, %	Myopathy	NYHA class	Arrhythmia	Conduction disturbance	Outcome
I.1	NA	NA			M	54	NA	NA	NA	NA	NA	NA	MI
I.2	NA	NA			F	64	NA	NA	NA	NA	NA	NA	alive
I.3	NA	NA			M	54	NA	NA	NA	NA	NA	NA	SCD
I.4	NA	NA			F	56	NA	NA	NA	NA	NA	NA	alive
II.1	NA	NA			F	41	NA	NA	NA	NA	NA	NA	alive
II.2	0	1			M	42	isolated LVNC with preserved EF	66	no	no	no	no	alive
II.3	1	0			F	38	healthy	65	no	no	no	no	alive
II.4	NA	NA			F	36	NA	NA	NA	NA	NA	NA	CHD
III.1	NA	NA			F	17	NA	NA	NA	NA	NA	NA	alive
III.2	NA	NA			M	14	NA	NA	NA	NA	NA	NA	alive
III.3	1	1			M	12	dilated LVNC	15	no	III	VT	no	alive

Pedigree of the index patient Fam022

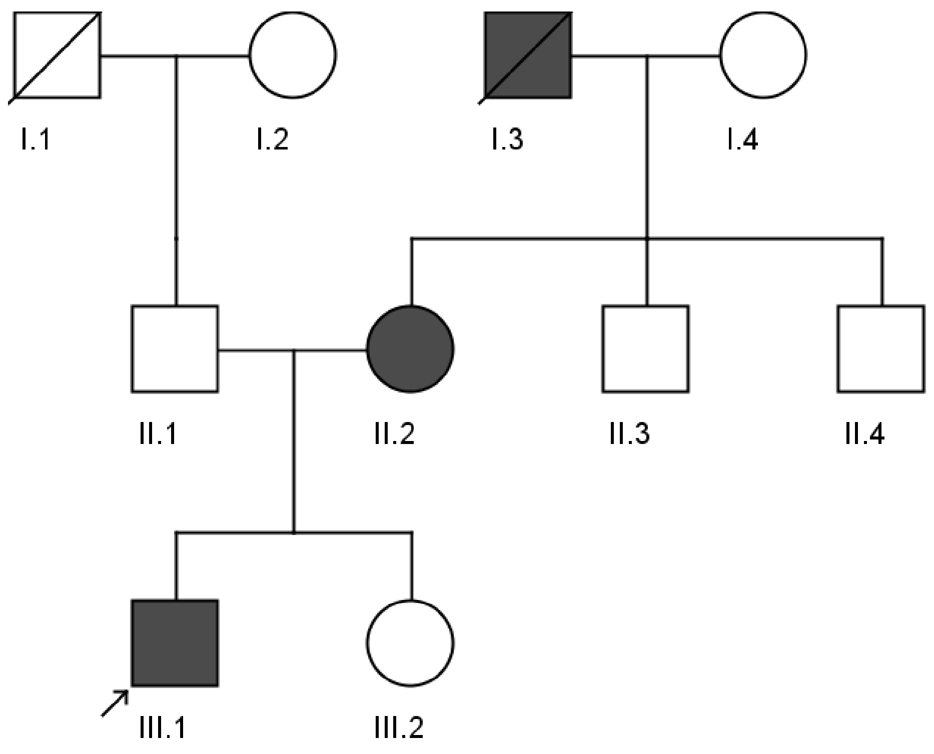

Patient ID	Genotype[1] *TTN*, p.Arg21639Ter	Genotype[1] *MYH7*, p.Arg1359Cys	Genotype[1] *DSC2*, p.Glu102Lys[2]	Genotype[1] *NEXN*, p.Glu470del[2]	Sex	Age, y.o.	LVNC subtype or other cardiomyopathy	EF, %	Myopathy	NYHA class	Arrhythmia	Conduction disturbance	Outcome
I.1	NA	NA	NA	NA	M	65	NA	NA	NA	NA	NA	NA	died (cancer)
I.2	NA	NA	NA	NA	F	67	NA	NA	NA	NA	NA	NA	alive
I.3	NA	NA	NA	NA	M	45	NA	NA	NA	NA	NA	NA	SCD
I.4	NA	NA	NA	NA	F	70	NA	NA	NA	NA	NA	NA	alive
II.1	NA	NA	NA	NA	M	45	NA	NA	NA	NA	NA	NA	NA
II.2	1	0	0	0	F	45	isolated LVNC with preserved EF	55	no	no	VE	no	alive
II.3	NA	NA	NA	NA	M	48	NA	NA	NA	NA	NA	NA	NA
II.4	0	0	0	0	M	42	healthy	64	no	no	no	no	alive
III.1	1	1	1	1	M	22	dilated LVNC	46	no	II	VT	no	alive
III.2	1	0	0	0	F	17	healthy	73	no	no	no	no	alive

Pedigree of the index patient Fam023

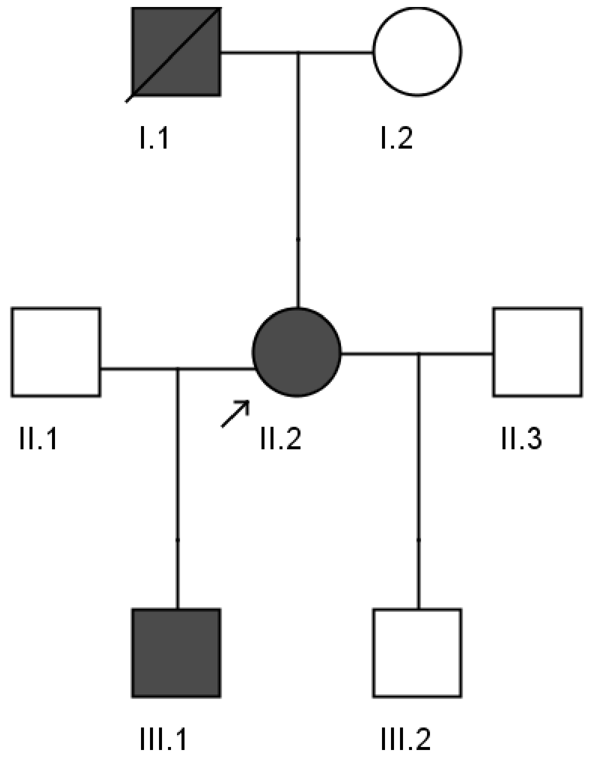

Patient ID	Genotype[1] *DES*, p.Ala337Pro	Genotype[1] *DSP*, p.Leu1348Ter	Genotype[1]	Genotype[1]	Sex	Age, y.o.	LVNC subtype or other cardiomyopathy	EF, %	Myopathy	NYHA class	Arrhythmia	Conduction disturbance	Outcome
I.1	NA	NA			M	47	DCM	NA	NA	NA	NA	NA	died (DCM)
I.2	0	0			F	68	healthy	62	no	no	no	no	alive
II.1	NA	NA			M	47	healthy	68	no	no	no	no	alive
II.2	1	1			F	44	dilated LVNC	28	+	III	AF, VT	LBBB	alive
II.3	NA	NA			M	46	healthy	56	no	no	no	no	alive
III.1	1	0			M	23	isolated LVNC with preserved EF	57	+	no	no	no	alive
III.2	0	1			M	18	healthy	68	no	no	no	no	alive

Pedigree of the index patient Fam024

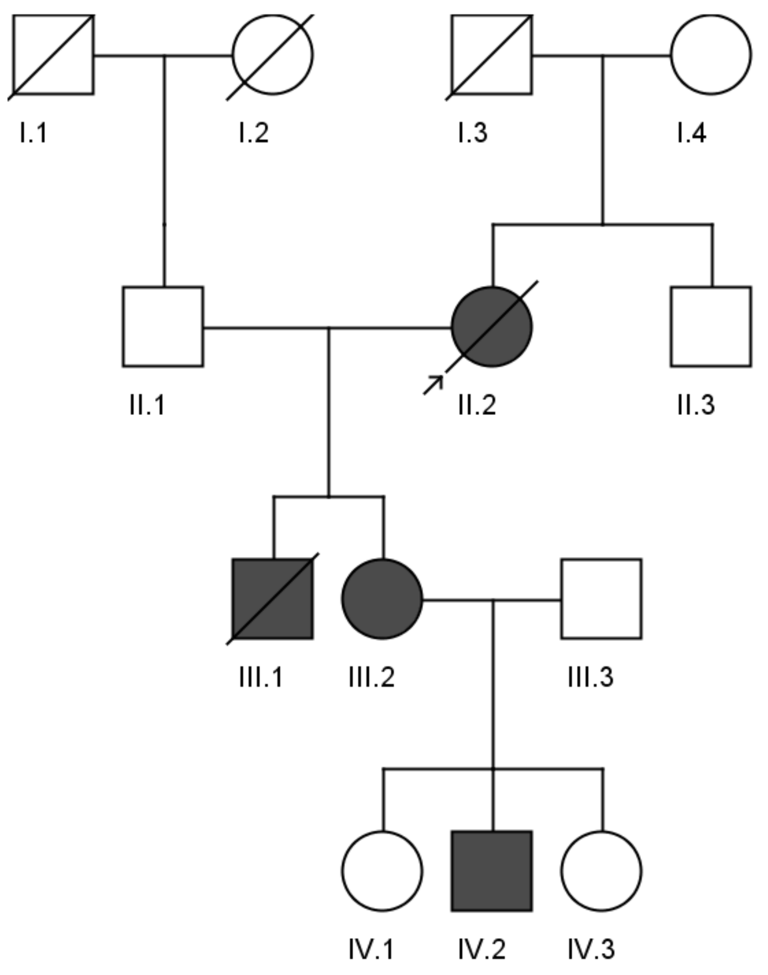

Patient ID	Genotype[1] *FBN2*, p.Asp2449Asn	Genotype[1] *PLEC*, p.Val3153AlafsTer77	Genotype[1]	Genotype[1]	Sex	Age, y.o.	LVNC subtype or other cardiomyopathy	EF, %	Myopathy	NYHA class	Arrhythmia	Conduction disturbance	Outcome
I.1	NA	NA			M	65	NA	NA	NA	NA	NA	NA	died (unknown)
I.2	NA	NA			F	64	NA	NA	NA	NA	NA	NA	died (unknown)
I.3	NA	NA			M	65	NA	NA	NA	NA	NA	NA	died (unknown)
I.4	NA	NA			F	75	NA	NA	NA	NA	NA	NA	died (unknown)
II.1	NA	NA			M	63	NA	NA	NA	NA	NA	NA	alive
II.2	1	1			F	57	dilated LVNC	18	no	III	VT	LBBB	CVD
II.3	NA	NA			M	51	NA	NA	NA	NA	NA	NA	NA
III.1	NA	NA			M	17	NA	54	+	no	no	no	died (myopathy)
III.2	1	1			F	36	isolated LVNC with preserved EF	55	no	no	VE	no	alive
III.3	NA	NA			M	no	NA	NA	NA	NA	NA	NA	alive
IV.1	NA	NA			F	14	NA	NA	NA	NA	NA	NA	alive
IV.2	1	0			M	13	isolated LVNC with preserved EF	64	+	no	no	no	alive
IV.3	NA	NA			F	6	NA	NA	NA	NA	NA	NA	alive

Pedigree of the index patient Fam031

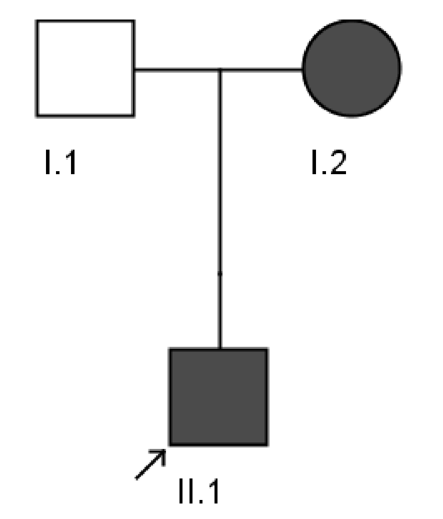

Patient ID	Genotype[1] *MIB1*, p.Tyr392Ter	Genotype[1] *MTMR14*, c.308+1G>A	Genotype[1] *MYH7*, p.Gly178Arg	Genotype[1]	Sex	Age, y.o.	LVNC subtype or other cardiomyopathy	EF, %	Myopathy	NYHA class	Arrhythmia	Conduction disturbance	Outcome
I.1	1	1	0		M	34	healthy	65	no	no	no	no	alive
I.2	0	0	0		F	30	isolated LVNC with preserved EF	54	no	no	VE	no	alive
II.1	1	1	1		M	4	isolated LVNC with reduced EF	71	no	no	VE	no	alive

Pedigree of the index patient Fam042

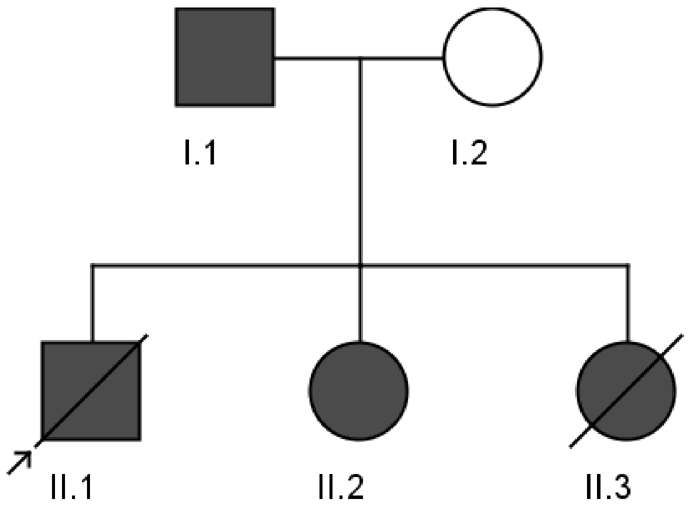

Patient ID	Genotype[1] *DSP*, p.Arg1452Ter	Genotype[1] *DSP*, p.Leu1669ThrfsTer15	Genotype[1]	Genotype[1]	Sex	Age, y.o.	LVNC subtype or other cardiomyopathy	EF, %	Myopathy	NYHA class	Arrhythmia	Conduction disturbance	Outcome
I.1	0	1			M	42	isolated LVNC with preserved EF	64	no	no	no	no	alive
I.2	1	0			F	34	healthy	63	no	no	no	no	alive
II.1	1	1			M	9	dilated LVNC	36	no	I	VE	LBBB	died (HF)
II.2	0	1			F	11	isolated LVNC with preserved EF	69	no	I	no	no	alive
II.3	NA	NA			F	6	DCM	NA	NA	NA	NA	NA	died (DCM)

Pedigree of the index patient Fam062

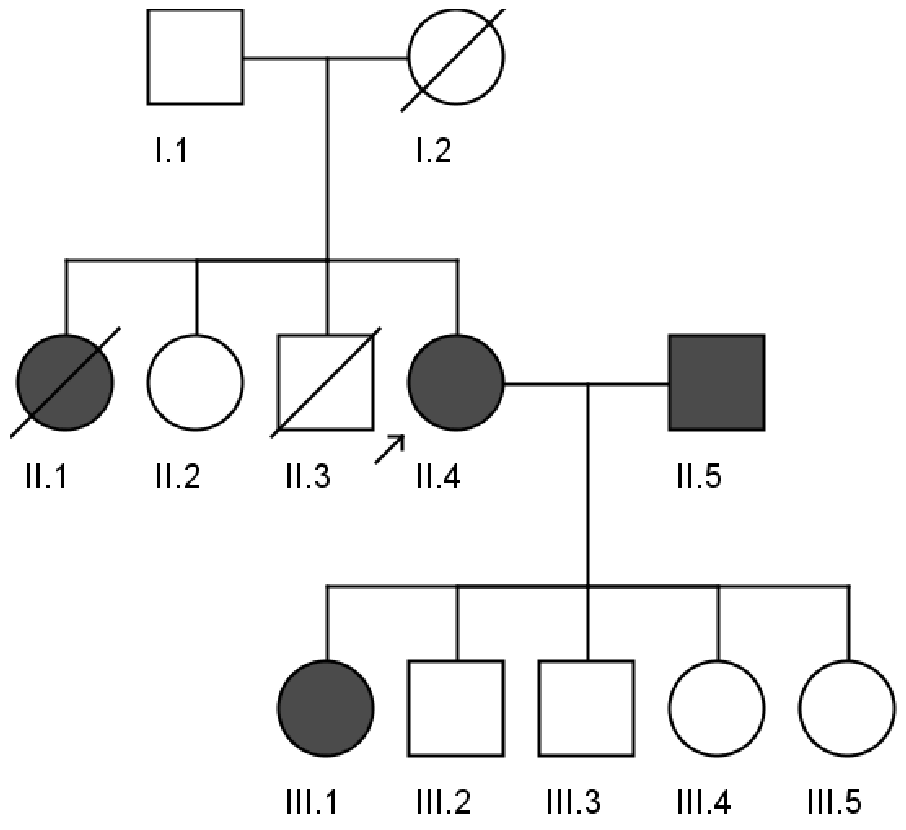

Patient ID	Genotype[1] *SLC22A5*, p.D388Ifs*11	Genotype [1] *MYH7*, p.Ala1632Thr	Genotype[1] *TTN*, p.Arg16391Ter	Genotype[1]	Sex	Age, y.o.	LVNC subtype or other cardiomyopathy	EF, %	Myopathy	NYHA class	Arrhythmia	Conduction disturbance	Outcome
I.1	NA	NA	NA		M	58	NA	NA	NA	NA	NA	NA	alive
I.2	NA	NA	NA		F	53	NA	NA	NA	NA	NA	NA	died
II.1	NA	NA	NA		F	34	DCM	NA	NA	NA	NA	NA	died (DCM)
II.2	NA	NA	NA		F	NA	NA	NA	NA	NA	NA	NA	alive
II.3	NA	NA	NA		M	18	NA	NA	NA	NA	NA	NA	died (accident)
II.4	1	1	1		F	30	isolated LVNC with reduced EF	45	no	II	no	no	alive
II.5	NA	NA	NA		M	33	DCM	42	no	III	AF (paroxismal)	no	alive
III.1	2	0	0		F	9	DCM	44	no	II	no	AVB	alive
III.2	NA	NA	NA		M	5	NA	NA	NA	NA	NA	NA	alive
III.3	NA	NA	NA		M	3	NA	NA	NA	NA	NA	NA	alive
III.4	NA	NA	NA		F	2	NA	NA	NA	NA	NA	NA	alive
III.5	NA	NA	NA		F	1	NA	NA	NA	NA	NA	NA	alive

Pedigree of the index patient Fam103

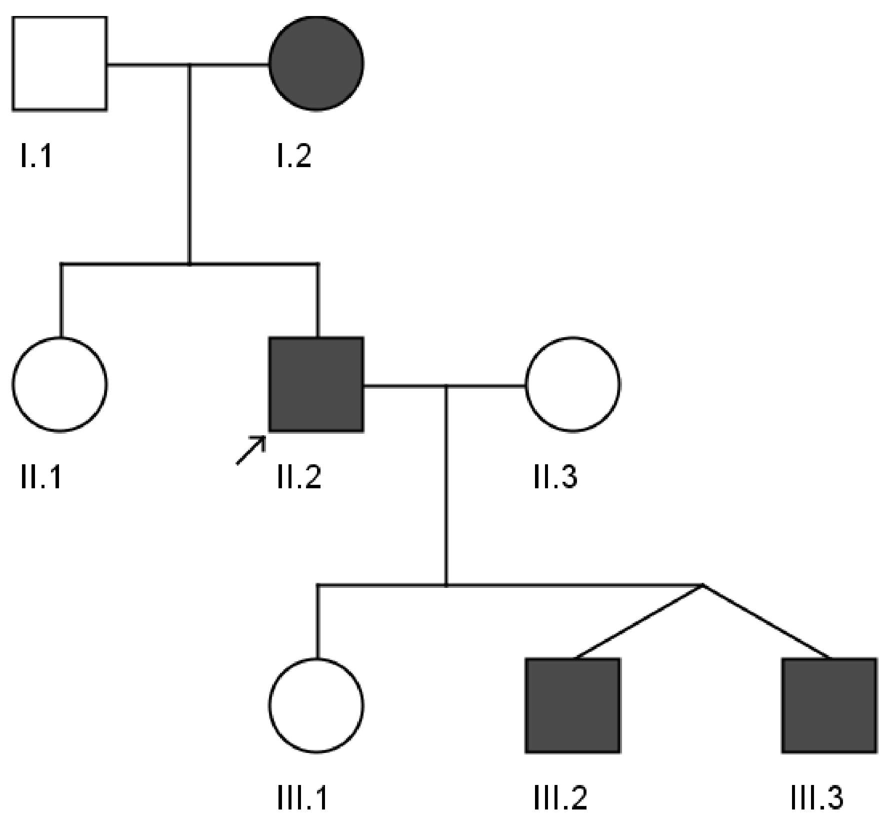

Patient ID	Genotype[1] *MYBPC3*, p.Arg597Gln	Genotype[1] *DSP*, c.273+5G>A[2]	Genotype[1]	Genotype[1]	Sex	Age, y.o.	LVNC subtype or other cardiomyopathy	EF, %	Myopathy	NYHA class	Arrhythmia	Conduction disturbance	Outcome
I.1	NA	NA			M	79	NA	NA	NA	NA	NA	NA	alive
I.2	1	0			F	76	HCM	63	no	II	AVB type 1	VE	alive
II.1	NA	NA			F	51	healthy	NA	no	no	no	no	alive
II.2	1	1			M	48	hypertrophicnodilated LVNC	46	no	III	AF (paroxismal)	no	alive
II.3	0	0			F	50	healthy	63	no	no	no	no	alive
III.1	0	0			F	25	healthy	68	no	no	no	no	alive
III.2	1	0			M	20	isolated LVNC with preserved EF	65	no	no	no	VE	alive
III.3	1	0			M	20	isolated LVNC with preserved EF	61	no	no	SAB	VE	alive

AF — atrial fibrillation; AVB — atrioventricular block; CVD — cardiovascular disease; DCM — dilated cardiomyopathy; EF — ejection fraction; HCM — hypertrophic cardiomyopathy; HF —heart failure; LBBB — left bundle branch block; LVNC — left ventricular noncompaction; MI — myocardial infarction; NA — data not available; NYHA — New York Heart Association; SAB — sinoatrial block; SCD — sudden cardiac death; VE — ventricular extrasystole; VT — ventricular tachycardia; VUS — variant of unknown significance; y.o — years old. "no" — no evidence of disease in the patient; "+" — disease confirmed.

[1] Genotype: 0 = wild type, 1 = heterozygous variant, 2 = homozygous variant.

[2] Rare VUS variants that can modify the cause of the disease.
